# Security and Privacy for Mobile IoT Applications Using Blockchain

**DOI:** 10.3390/s21175931

**Published:** 2021-09-03

**Authors:** Kevin Carvalho, Jorge Granjal

**Affiliations:** Centre for Informatics and Systems, University of Coimbra, 3030-290 Coimbra, Portugal; jgranjal@dei.uc.pt

**Keywords:** IoT, privacy, security, location, blockchain, MQTT

## Abstract

Internet of Things (IoT) applications are becoming more integrated into our society and daily lives, although many of them can expose the user to threats against their privacy. Therefore, we find that it is crucial to address the privacy requirements of most of such applications and develop solutions that implement, as far as possible, privacy by design in order to mitigate relevant threats. While in the literature we may find innovative proposals to enhance the privacy of IoT applications, many of those only focus on the edge layer. On the other hand, privacy by design approaches are required throughout the whole system (e.g., at the cloud layer), in order to guarantee robust solutions to privacy in IoT. With this in mind, we propose an architecture that leverages the properties of blockchain, integrated with other technologies, to address security and privacy in the context of IoT applications. The main focus of our proposal is to enhance the privacy of the users and their data, using the anonymisation properties of blockchain to implement user-controlled privacy. We consider an IoT application with mobility for smart vehicles as our usage case, which allows us to implement and experimentally evaluate the proposed architecture and mechanisms as a proof of concept. In this application, data related to the user’s identity and location needs to be shared with security and privacy. Our proposal was implemented and experimentally validated in light of fundamental privacy and security requirements, as well as its performance. We found it to be a viable approach to security and privacy in IoT environments.

## 1. Introduction

Internet of Things (IoT) covers a comprehensive range of applications that fall within various areas of our daily lives. These applications intend to make our personal or work-related tasks easier by generating a multitude of data that can be processed for various purposes, ranging from automation to data analysis. Despite the various benefits these applications offer when being part of our daily lives, privacy concerns emerge in various situations. These concerns are mostly related to the generation of privacy-sensitive information, which can be prone to leakages and lead to potential attacks. The data transmitted and processed by devices supporting such applications can frequently contain personal details, and as a result, the user should be able to control their exposure. In fact, user-controlled data exposure and privacy is becoming increasingly important and even legally imposed worldwide, as it is the case with requirements identified in the General Data Protection Regulation (GDPR) [1] legislated by the European Union (EU).

The expansion of IoT does not seem to stagnate soon. According to the International Data Corporation (IDC), it is estimated that 55.7 billion IoT devices will be connected worldwide in 2025 [2], generating 73.1 zettabytes (ZB) of data. Therefore, it is important to develop robust solutions that preserve the privacy of the users’ identity and allow them to control the exposure and usage of their data. Existing approaches to security and privacy in IoT involve techniques such as obfuscation [3,4] and one-time passwords (OTP) [5], but mostly focus on the privacy of the data, and not on the privacy or the exposure of the user himself. Furthermore, another important aspect is to guarantee the privacy of the data while it is stored and processed in the underlying systems and devices supporting the application. Thus, the solutions should not only focus on the edge layer, but also on the cloud layer, where the data is exchanged, stored and processed in the context of the IoT application. In this context, we may identify blockchain as a promising technology, which can be explored to enhance the privacy of the users [6]. Since users can remain anonymous in this system, information can be shared without disclosing the owner’s identity, while we may note that may not apply to every decentralised application, since some of those need to employ mechanisms such as Know Your Customer (KYC) and Anti-Money Laundering (AML) for security purposes.

In contrast with classical architectures where a centralised trusted authority is employed, blockchain is a decentralised system, which makes it less vulnerable to single points of failure. The participating nodes are responsible for its proper functioning and validation, making it a network with immunity properties that is able to mitigate malicious actions autonomously (e.g., information tamper). In essence, the blockchain functions as a ledger that stores information (transactions) in blocks connected in a sequential format, resembling a chain. This feature allows exchanging crypto assets between users, and it also registers and keeps track of the blockchain’s state (e.g., how many crypto assets each entity possesses) securely and with high tamper resilience, thus also providing non-repudiation properties. Moreover, depending on the implementation, blockchain can also be employed to support user privacy, by providing anonymity. Despite all its benefits, this technology would not be as relevant if it was not applicable in contexts other than the exchange of crypto assets. In fact, one of its most important features is the possibility to create distributed applications through smart contracts, as we explore in our proposed architecture. This allows the mitigation of some risks inherent in centralised architectures and human-controlled systems, e.g., single points of failure, data loss, human errors and data corruption.

Some proposals in the literature take advantage of blockchain technology to enhance the privacy of IoT applications [4,7,8,9,10], although we verify that such works do not address all the core aspects of user privacy. We build on such proposals while addressing open issues related to security and privacy. Other than blockchain, we also consider the integration of other technologies to complement the functionalities provided by the proposed architecture. Among such technologies employed to mitigate particular limitations, we note the usage of the Message Queue Telemetry Transport (MQTT) [11] protocol for lightweight and resilient communications [12], and of Storj [13] for decentralised cloud storage. The main focus of our proposal is to implement and validate a proof of concept that employs such technologies, to enhance the users’ privacy and security in IoT applications, and also to enhance the interoperability between constrained IoT devices and the blockchain. To achieve such goals, the proposed architecture employs anonymisation that conceals the user’s identity and their data throughout the whole system, as well as user-controlled privacy allowing the user to configure the level of exposure of their data (e.g., which data type is shared and with whom), together with other classical security mechanisms (e.g., cryptography and access control). In addition, we consider an example application for which the proposed architecture could address security and privacy: a smart vehicle application in which data is shared, stored and exchanged in the system. In this application, our proposal helps in addressing security and privacy, particularly in concealing geolocation information generated by mobile users.

The paper is organised as follows. In Section 2, we identify and discuss the main requirements related to security and privacy that motivate our proposal. In Section 3, we discuss the proposed architecture and its main functionalities, which are evaluated in Section 4, particularly in relation to security, privacy and performance. Section 5 discusses related work and, finally, in Section 6, we address future research opportunities.

## 2. Background and Motivation

In our following discussion, we identify the main security and privacy issues and requirements in IoT, with a particular focus on applications requiring mobility. After that, we proceed to discuss the proposed architecture and the security and privacy mechanisms employed in its context.

### 2.1. Privacy and Security in IoT

Many of the current and envisioned IoT applications generate many security privacy concerns, in particular, due to the amount of sensitive data that is generated, transmitted, processed and finally stored in the context of such applications. In reality, we may realise that privacy concerns apply to applications with very different goals and contexts, in areas such as health care [14], smart parking [15,16] and smart vehicles [12,17,18], among others. In our proposal, we focus in particular on IoT applications in the context of which mobility is a requirement, and geolocation data is shared by the user along with the goals of the application, in the smart vehicles’ sector.

In itself, privacy is a concept which needs to be precisely defined in order to contextualise a particular approach and application context. In our proposal, we address the privacy of the user, which is related to the necessity of sharing different types of data, such as telemetry, location and personal user information. Therefore, it is imperative to materialise strategies to safeguard the sharing of such data, otherwise the user, as well as the application itself, may be subjected to various threats. Among such threats, we may find user tracking (e.g., obtaining a person’s daily activity history) and user profiling. Cryptography [4,7,8,10,19] and access control [3,4,5,19] mechanisms are commonly implemented to improve the privacy of the data. However, the identity of the users is not always concealed in such solutions.

Focusing now on applications that require mobility and precise location, and in particular, in the smart vehicles’ sector, we can observe that the data generated and exchanged can put the identity of the user at stake if its disclosure is not precisely controlled. Thus, robust privacy-preserving solutions are required to deal with all the aforementioned threats. Current data storage and exchange solutions are typically centralised and as such represent single points of failure, which are prone to data tamper and service downtime, among other threats. Furthermore, the privacy of the users’ identity and the data is not guaranteed in many of such approaches and, hence, the need to implement solutions that enhance privacy by design arises. With this in mind, our motivation is to consider the adoption of blockchain as a central component of our architecture to address security and privacy. Blockchain offers a distributed and decentralised ledger and helps in addressing some limitations observed in centralised approaches. In addition, blockchain supports privacy by design properties that allow its users to remain anonymous, since no sensitive information is required to create an identity in the blockchain [6,17,18].

In line with the previously discussed privacy requirements, security also presents fundamental enabling factors for the majority of IoT applications. This is also the case in the context of the proposed architecture, as we detail later in the article since we enable encryption for the data transported with MQTT and stored in the blockchain. Access control is also necessary since it allows enforcing access rules according to the role of the user in the context of the application, and, in this context, we explore the usage of smart contracts. Authentication and authorisation are also implemented based on the blockchain and MQTT. Finally, we note the importance of resilience, and, in this case, we explore the decentralised nature of the blockchain, as well as the usage of decentralised storage mechanisms.

We proceed by discussing the application considered for the identification of the security and privacy requirements that motivate our proposal. This application also guides our experimental evaluation of the proposed mechanisms, as we address later in the article.

### 2.2. Use Case

In order to contextualise the proposed architecture, as well as to identify its critical security and privacy requirements, we consider a crowd-sensing IoT mobile application, in the context of which smart vehicles (and its users) share information about road conditions and road events, which need to be shared with the other parties involved. The main goal of our proposal is to offer a system in which the data generated by such entities is stored and shared privately and securely. Thus, the data subjects (vehicle user) identity needs to remain anonymous, since we consider that no details are required or disclosed about the source of the information. The user should also be able to control the level of exposure of their data through an access control list (ACL) mechanism, in which the authorised end users (e.g., service providers and authorities) are identified.

We consider that, by default, vehicle users are required to define ACL, as appropriate to their requirements in terms of sharing data. Thus, in the first setup phase, an ACL needs to be defined and stored. This definition triggers the creation of a secret key that is shared with the authorised nodes. After the conclusion of this setup phase, data transmission can start. In the considered application, a smart vehicle’s image recognition system can detect a relevant event on the road, for example, an accident. In this situation, the event can be appropriately tagged, in a way that, when data related to the event is shared, only the nodes present in the corresponding ACL of that data type are able to access the data. This strategy can thus be used to guarantee that only certified authorities, emergency service nodes or other entities can access specific data, as intended. In addition, the shared data can contain coordinates to locate the incident and more relevant information as defined by the application at hand (e.g., the number of cars involved and the level of severity).

Another important aspect of the considered application is that shared data needs to be kept confidential. With this goal in mind, we employ symmetric encryption with pre-shared secret keys. When the shared data is stored, a log event is broadcast to the public, which contains information about the data type and other non-sensitive details. For example, the authority’s agent node parses the event and checks that it is a data type of interest. The agent node can then make a data request which, after authorisation, gives them access (after decryption) to data details for internal system purposes (e.g., to dispatch a team to the location of the accident). We detail the previously described operational procedures and requirements next, in the context of our discussion on the functionalities and technological decisions of the proposed architecture.

### 2.3. Functionalities and Technological Decisions

The next subsection identifies the main functionalities and technological decisions considered in the operation and implementation of the proposed architecture, in order to cope with the previously identified security and privacy requirements.

#### 2.3.1. Anonymisation and Information Exchange Using Blockchain

As previously discussed, we consider the usage of blockchain [20] to anonymise the identity of the user and their data. Furthermore, we also employ blockchain as a decentralised system to store and exchange non-sensitive information, as well as to store the privacy preferences of users (as defined by ACL) to ultimately perform access control through smart contracts. The actual vehicle users’ data is stored off-chain in Storj, as detailed later. Storj mitigates some of the inherent problems of centralised systems, such as single points of failure, the possibility of information tampering and centralised governance. In addition, we implement anonymisation of users using the addressing system of blockchains, which by design enhances the privacy of the users’ identity, since no sensitive information is attached to their address. With this approach, the user is identified by their blockchain address throughout the whole system, whether in MQTT, Storj or smart contracts.

Blockchains may be divided into two categories: permissionless and permissioned blockchains. Permissionless blockchains, as the name suggests, allow any node to participate and interact with it. In contrast, permissioned blockchains can impose restrictions on the nodes for the access, such as mining or validating blocks and, in addition, employ a trustful approach, meaning that nodes are considered trustworthy only if properly authorised. The security of this system depends on the correct operation of the consensus protocol implemented, which dictates how a blockchain’s state is maintained and verified. The most popular blockchains use Proof of Work (PoW), currently used by Bitcoin [21] and Ethereum [22], and Proof of Stake (PoS), used in Cardano [23] and, in the future, in Ethereum 2.0 [24]. On the other hand, smart contracts [20] allow to extend the functionalities and applicability of blockchains. Smart contracts extend the functionalities supported by the blockchain, in fact enabling automated, secure, and tamper resilient tasks (e.g., controlling access to a certain function).

In conclusion, blockchain is used to anonymise the identity of the user and also as a medium to store and exchange non-sensitive information and to support access control to data requests, through smart contracts. The tamper resilience and transparency features are interesting for applications where privacy and data management are not relevant, contrary to the integrity of the stored data. Therefore, storing sensitive information in the blockchain is not an option, even if a strong cryptographic algorithm is in place.

#### 2.3.2. Lightweight Communications Using MQTT

In order to provide appropriate support for lightweight and resilient communications in the context of the proposed architecture, we adopt the MQTT [25] communications protocol. The main goal in this context is to promote resource savings in constrained environments and devices, by not requiring the support of blockchain in such nodes. The support of blockchain in devices with resource constraints is a well-known problem since its support is recognised to be resource-intensive [6]. MQTT is typically used in IoT applications, due to its small footprint in terms of the size of the messages employed, as well as its asynchronous nature. MQTT uses a publish and subscribe model that stores messages in topics to be transmitted to MQTT subscribers. It uses Transmission Control Protocol (TCP) as the transport protocol, which adds reliability to communications. It also provides resilience mechanisms, such as durable connections and Quality of Service (QoS), which are advantageous for applications with volatile environments (e.g., blind spots and signal degradation), as may be the case in smart vehicles. Therefore, MQTT facilitates the enabling of failover and reliability properties. Furthermore, MQTT enhances the interoperability between constrained devices that do not support blockchain.

#### 2.3.3. Decentralised Cloud Storage

The tamper resilient and transparent nature of blockchains makes them unsuitable for storing sensitive information, especially user data. With this aspect in mind, we employ an off-chain storage solution in the proposed approach. More precisely, Storj [13] has been employed for this purpose. This storage system is used to store the vehicle users’ data, and it allows for deletion and modification of data on-demand, an important aspect to promote the compliance of our proposal with requirements related to user-controlled privacy and data exposure. Storj is a decentralised and S3-compatible storage platform, implementing default data encryption using AES-256-GCM, as well as redundancy and resilience. Using Storj, data is stored as individual fragments distributed throughout various servers, in a globally decentralised network. Thus, it prevents data breaches and provides resilience to data loss, since only a percentage of those fragments are needed to reconstruct data in case of necessity. Data stored is accessible via a serialised interface, identified by a string that can be used in the uplink application or defined for using the libuplink library. It is this string that we store in the blockchain and exchange with authorised nodes. In the next section, we discuss, in greater detail, the functionalities implemented by the blockchain in the context of the proposed architecture.

## 3. An Architecture for Security and User-Controlled Privacy in Mobile IoT Applications

### 3.1. Proposed Architecture

**Figure 1:**
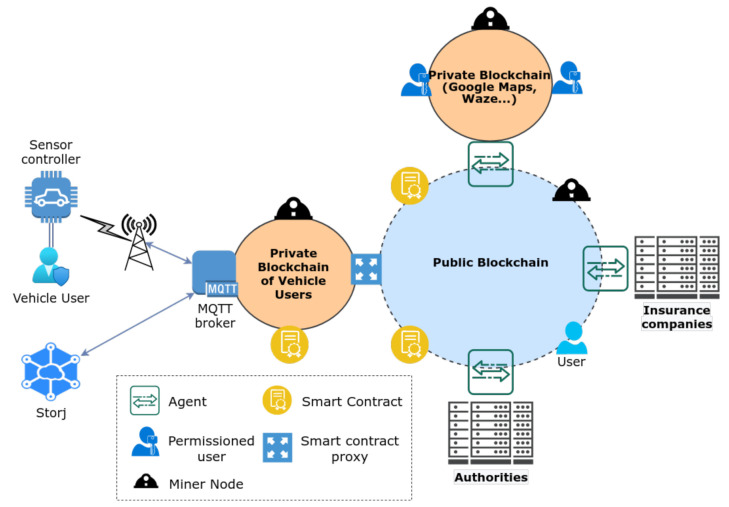
An architecture for security and privacy in mobile IoT applications.

We start by presenting the proposed architecture, which has been designed to cope with the previously discussed requirements. The components that enable the architecture illustrated in Figure 1 support the various functionalities required to enable storage, management and exchange of data produced by vehicle users with security and privacy, among others. The main technological components employed are blockchain, MQTT and Storj. Blockchain functions as a medium to store non-sensitive information (such as ACL) and metadata (e.g., related with access requests), and to exchange information. Since blockchain is tamper resilient, the information is not easily modified. MQTT is used as a lightweight communication protocol and a middleware between the constrained IoT devices and the blockchain core. On the other hand, Storj is the storage system employed to store sensitive data. In the next section, we provide a more in-depth overview of these technologies, together with the motivation for their employment in the context of our architecture.

The main requirements considered in the design of our proposal are focused on providing enhanced privacy and security to IoT with mobility, and we must note in particular the following:

- User anonymity: the system focuses on providing privacy to the vehicle user’s identity through the use of blockchain, given that the blockchain account of each vehicle user cannot be correlated with their personal information. Furthermore, the blockchain address of the user is only used for identification purposes in the Private Blockchain of Vehicle Users (PBVU). Therefore, linking attacks can be prevented.
- Location and data privacy: inherently from the user’s anonymity, data generated by users is anonymous. Therefore, the privacy of the user is enhanced, which prevents profiling and tracking attacks.
- User-controlled privacy: vehicle users have to define a specific list of nodes that are authorised to access their data. Each type of data (e.g., related to an accident, road or traffic conditions) possesses an ACL that defines the rules for controlling accesses.
- Data confidentiality: security and privacy of the data in all processes where data is used and transmitted is enforced via symmetric encryption.
- Authentication and Authorisation: provide authentication of vehicle users when communicating with the MQTT broker to send requests, and restrict their operations to enhance each vehicle user’s data security.

We process our discussion by analysing in detail the operation and the technological aspects of the various components enabling the proposed architecture, as well as their role in fulfilling the aforementioned requirements.

### 3.2. A Blockchain Tiered Architecture

In the architecture illustrated in Figure 1, we employ two types of blockchains: public and private blockchains (PBVU). The two blockchains are connected via a bridge device, in particular a smart contract proxy. This approach was an architectural decision to enhance the security of the system, since using only one public or private blockchain would have limitations in terms of security and accessibility. With this approach, each blockchain has a different and complementary goal, which takes advantage of their benefits, improves privacy and security of certain information (e.g., privacy preferences and data serials), decentralises the system even more to reduce congestion, and enhances scalability.

The PBVU is a closed and permissioned blockchain offering more privacy and security when compared to permissionless blockchains, and as a result it is suitable to store information that should not be available publicly. Therefore, in our architecture, this chain stores serialised access strings of data to be shared with authorised public nodes, and the users’ access control lists (ACL) to perform access control in its smart contracts. The public blockchain is a permissionless blockchain (open to the public) that advertises information to the public nodes and relays requests to the private blockchain through its smart contracts and the smart contract proxy. The bridging nodes participate in both types of systems to handle requests and allow intermediation. They are:

- A smart contracts proxy: this entity is responsible for interconnecting the PBVU with the public blockchain. It listens and handles requests made in both blockchains. For security reasons, this node only handles requests created by smart contracts to prevent the forging of requests by malicious nodes.
- The agent: this node is managed by entities (e.g., governments or companies using the application), it connects the entity’s systems to the public blockchain, whether it is a private blockchain or a more centralised infrastructure. Its main purpose is to make data requests and retrieve the data to the infrastructure.

The public blockchain, as the name implies, uses a permissionless network that is already deployed and available, in particular, the mainnet of Ethereum [22] or Cardano [23]. Thus, the nodes can easily participate in the system. The PBVU is a permissioned blockchain that is formed by a set of trusted high-level nodes, which can be MQTT brokers, smart contract proxies and other types of nodes.

The blockchain protocol selected for our solution was Ethereum [22], a mature blockchain that provides a wide range of functionalities, such as smart contracts, creation and configuration of private networks and testnets (Ethereum-based public blockchains for tests [26]). We proceed to discuss the usage of smart contracts in the context of the architecture illustrated in Figure 1.

### 3.3. Smart Contracts

Smart contracts are the essential components that allow other devices to interact with the system, in order to execute certain system processes. Each system’s blockchain type has a smart contract designed with different purposes, and consequently possessing a different set of functions. The main functionalities they provide to this system are storing information in-chain and broadcasting events to interact with the software in the nodes.

Smart contracts are deployed in the blockchains with a specific blockchain address and are responsible for executing a certain number of programmed functions. The functions available to be called by the blockchain nodes are:

1. *defineACL*: receives as parameters the owner address, the data type and the list of blockchain addresses of the public blockchain nodes authorised to receive data of this type, which each vehicle user generates. When executed, this function creates a transaction and stores the list of authorised blockchain addresses in the PBVU.
2. *exchangeKey*: receives the symmetric key encrypted with a public key of an authorised node as a parameter. This function is responsible for requesting the creation of an event on the public blockchain, addressed to the node allowed to decrypt the symmetric key. A request is sent to the smart contract proxy to create this event, which executes the *setKey* function to generate it in the public blockchain.
3. *addData*: this function receives the data information (owner address, data type and ID) and the serial of the data. Since the MQTT broker previously authenticated the blockchain address of the owner, the smart contract does not need to check that he is authorised to add data. Then, it proceeds to store the data, which generates a transaction inside the PBVU and creates an event to request the proxy node to generate log information about the data in the public blockchain.
4. *setLog*: receives the information about newly stored data, sent by the proxy nodes, to create a log event. The public blockchain nodes use the log events to fetch information about the data, including its ID, to make access requests.
5. *setKey*: is responsible for generating the event to broadcast the encrypted symmetric key in the public blockchain to be parsed by the destination node. It receives the address of the destined public node and the encrypted symmetric key.
6. *getPrivData*: receives the ID of the data requested by the public blockchain node as a parameter. This function also receives a specific amount of cryptocurrency, as a fee to pay for the subsequent transaction needed to request the data and prevent request flooding from public nodes. Next, a request is made to the proxy as an event to request the data to the PBVU’s smart contract.
7. *getData*: this function is executed by the smart contract proxy, which receives the ID of the data and the blockchain address of the requester as a parameter. Next, if the node is authorised to receive the data, its serial is sent back to the proxy, which forwards it to the public blockchain’s smart contract by executing the *setNewDataLog* function.
8. *setNewDataLog*: this function sends the *get data* request response as an event to the public blockchain, addressed to the data requester.

We employ two types of smart contracts in the proposed architecture: PBVU’s smart contract and public blockchain smart contract. The former is responsible for storing the serials of the vehicle users’ data, exchanging secret keys, managing access control and responding to data requests from the public blockchain (functions number 1, 2, 3 and 7 in the previous list). On the other hand, the latter receives access requests from public nodes (users and agents) and requests to broadcast events (functions 4, 5, 6 and 8).

### 3.4. Request Handlers

As Figure 1 illustrates, the proposed architecture comprises various intermediary nodes, namely the MQTT broker, smart contract proxy and agent. Each of these nodes contains software that awaits events or requests to be handled. Contrary to the other handlers, the MQTT broker does not process events broadcasted in the blockchain. Instead, it listens to MQTT messages sent by the sensor controller (a device responsible for sending the data generated in the vehicle). These messages contain specific commands that instruct what actions the broker shall perform.

The system’s requests are composed of a dictionary with three keys. These keys can change if the request is sent in-chain (blockchain event) or off-chain (MQTT messages). The common keys are command (identifies the command) and dataInfo (data information). The third key is *destinationAddr*, which may optionally be used to identify the destination blockchain address in in-chain requests, or value, used for encrypted data or other information in off-chain requests.

### 3.5. Symmetric and Asymmetric Cryptography

Symmetric and asymmetric encryption algorithms are conjugated to guarantee data confidentiality. On the one hand, the data is encrypted using a symmetric key, which is pre-shared via the blockchain, as previously discussed. Each vehicle user’s data type has a unique symmetric key and only the nodes authorised by an ACL are allowed to access this key. On the other hand, asymmetric encryption is used to exchange the key securely, with the symmetric key being encrypted with the public key of each authorised node. Therefore, only the node with the corresponding private key has access to the shared symmetric key used for encryption of communications. Regarding symmetric cryptography, we employ the Advanced Encryption Standard (AES) [27] with 256-bit keys, and Transport Layer Security (TLS) is enabled for MQTT communications.

In order to take advantage of the underlying cryptographic scheme provided by Ethereum, we use the Elliptic Curve Integrated Encryption Scheme (ECIES) framework with secp256k1 [28]. Since the key pair of each account created in Ethereum uses Elliptic Curve Digital Signature Algorithm (ECDSA) with the secp256k1 algorithm [22,28], they can be leveraged to use ECIES. This way, the symmetric key can be encrypted and exchanged securely to the authorised nodes, using their public key.

### 3.6. Processes and Interactions

**Figure 2:**
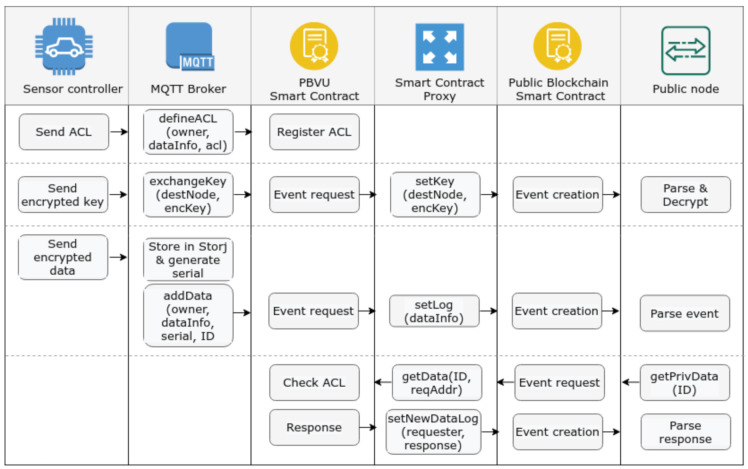
Overview of the system’s processes.

**Figure 3:**
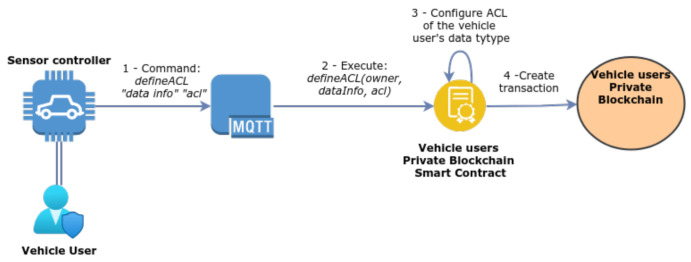
Vehicle user’s ACL definition.

**Figure 4:**
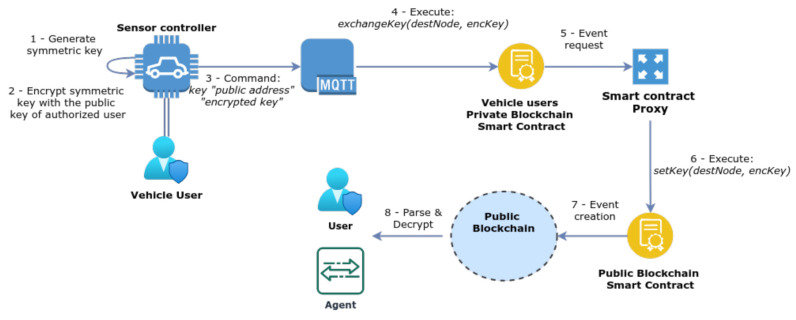
Exchange of the symmetric key with authorised users.

**Figure 5:**
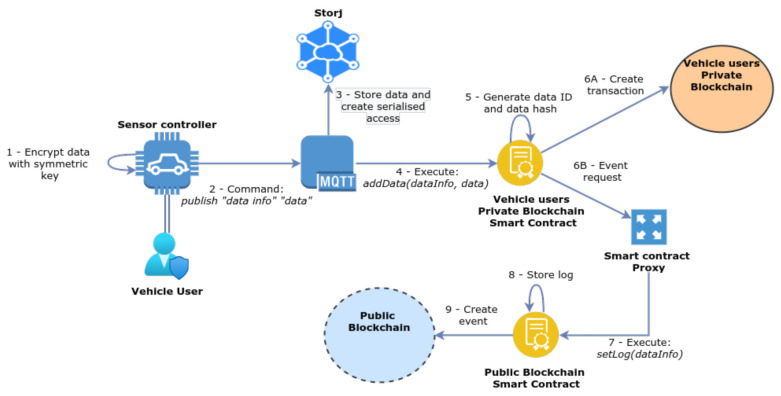
Data published by a sensor controller to be stored in Storj and in the blockchain.

**Figure 6:**
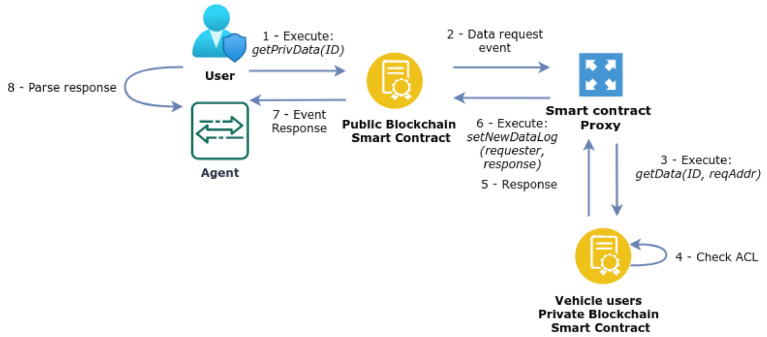
Data access request by an authorised node of the public blockchain.

**Figure 7:**
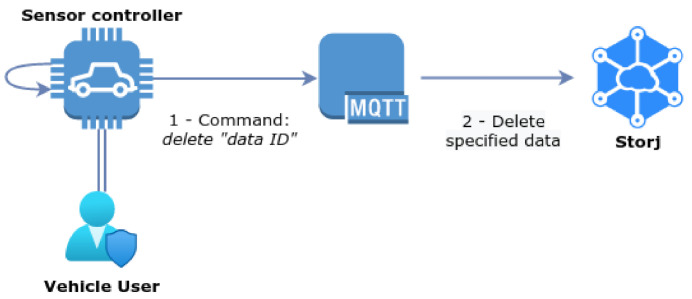
Data erasure request.

We find it important to analyse in greater detail how the various components of the proposed architecture operate, in order to materialise the functionalities defined in line with the requirements previously discussed. Figure 2 illustrates an overview of the various processes involved, which are detailed in Figure 2, Figure 3, Figure 4, Figure 5, Figure 6 and Figure 7 and discussed next, starting with the initial phase of ACL definition by the end-user of the application.

#### 3.6.1. Definition of Access Control Rules

Figure 3 illustrates how the vehicle user may define a list of users that can receive their data. This process begins in the sensor controller, which sends an MQTT *publish* message to the vehicle user’s topic, and, as mentioned before, these messages contain a dictionary to identify the request type and other relevant information. In this case, the message is formed by the following fields: *defineACL “data type” “ACL”*. The ACL is sent in the clear because it is assumed that the encryption enabled for MQTT communications provides adequate confidentiality. When successfully sent, the MQTT broker fetches the message and proceeds to handle the request. Next, the MQTT broker executes a function available from the PBVU’s smart contract, which is *defineACL (owner, dataInfo, acl)*. Finally, the smart contract stores this information in the vehicle user’s privacy preferences.

#### 3.6.2. Key Exchange

The definition of an ACL triggers another process, which is the symmetric key exchange with the nodes defined in the ACL. The secret key is generated in the sensor controller as a unique 256-bit key, and it is encrypted using the public key of the destination authorised node. As illustrated in Figure 4, the sensor controller sends the encrypted pre-generated key to the MQTT broker as a message that is composed of the fields *key “blockchain address” “encryptedKey”*. This message instructs the MQTT broker to execute the smart contract’s function *exchangeKey (destNode, encKey)*. When executed, the smart contract creates a request for the proxy to create an event in the public blockchain, addressed to the authorised node. This process is executed every time a new ACL is defined by the vehicle user, to renew the symmetric key when new nodes are added or removed from the list.

#### 3.6.3. Publishing and Storing Data

Figure 5 illustrates the process of sharing data, in which the sensor controller sends the data to be stored and exchanged in the system. Similarly to the previous processes, the data transmitted by the sensor controller is transported in a message addressed to the MQTT topic to which the user (vehicle) is subscribed. Before sending the message, data is encrypted with the corresponding pre-shared symmetric secret key, and the message contains the values *publish “data type” “encrypted data”*, which instruct the MQTT broker to store the data using Storj, and generate a serialised access string for the new data entry. This serial is what is stored in-chain and, for this purpose, the MQTT broker executes the function *addData (owner, dataInfo, serial, ID)* defined in the smart contract. The *ID* is a Universally Unique IDentifier (UUID) generated by the broker for each data entry stored in the distributed database. The smart contract stores the serial and generates an event to request the proxy to create a log event in the public blockchain, as previously discussed. This log event broadcasts information about the newly added data, such as the *ID* and the data type.

#### 3.6.4. Data Access Requests

One operation also required in the context of the proposed architecture is when a public node (an agent or a user device) requests access to a particular data record stored in the PBVU, as illustrated in Figure 6. To support this type of request, it executes the *getPrivData(ID)* function of the smart contract. Next, the smart contract generates an event to request the data serial from the smart contract proxy, and in consequence, the proxy executes the *getData (reqAddr, ID)* function of the smart contract in the PBVU. This function performs the access control check for the node request and, if the node is authorised in the ACL, the serial of the requested data is retrieved by the proxy, which executes the *setNewDataLog (requester, response)* function, again defined in the smart contract of the public blockchain. This function retrieves the serial to the requester node as an event. Finally, the requester is able to decrypt the received data using the pre-shared symmetric key.

#### 3.6.5. Data Management

The final process considered in the context of our architecture is related to on-demand management procedures that the vehicle user can perform over their data. Similarly to the previous processes, the vehicle user can request the broker to access, delete or modify a previously shared data entry, through commands sent in MQTT *publish* messages. It is important to note that only the owners of the data entry can perform such actions, which is guaranteed using the authorisation features of MQTT. Such mechanisms are also important to implement the aforementioned **security requirements**, in particular for the enabling of control of the user over their data. Figure 7 illustrates the data erasure procedure performed by a vehicle user, **to erase** one of their data entries identified by the corresponding ID. In the next section, we proceed by discussing in detail the experimental implementation and evaluation of the proposed system architecture, in the light of the previously identified security and privacy requirements.

## 4. Experimental Evaluation of the Proposed Architecture

We begin by addressing the experimental scenario considered for the implementation and evaluation of the proposed mechanisms, after which we address the evaluation metrics and analyse the obtained results.

### 4.1. Experimental Scenario and Test Conditions

**Figure 8:**
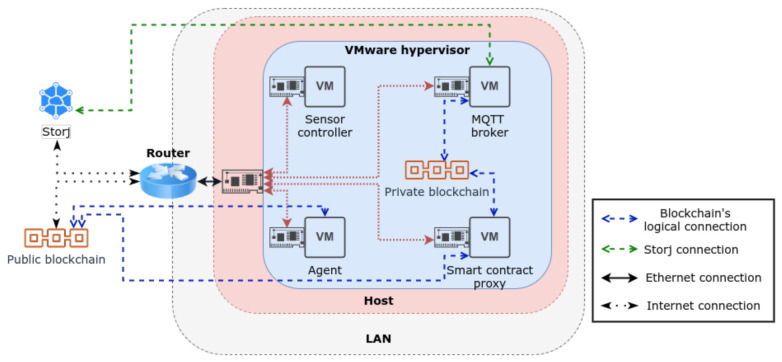
System architecture of the experimental evaluation scenario.

The experimental scenario is illustrated in Figure 8, where we consider the various technologies employed, as well as the logical and physical connections used to support communications between the various entities involved. In broad terms, we can observe that the components of the architecture consist of one physical machine hosting 4 virtual machines (VM), one local area network supporting 1 Gbps Ethernet communications between the router and the host machine, and a 200 Mbps uplink to the internet for communications with the public blockchain and Storj distributed storage service.

The host machine is powered by an i7-7700HQ 8-core processor running at 2.80 GHz and 16 Gb of RAM. We use Linux Ubuntu 16.04 in each VM in runlevel 3, in order to save resources. We now analyse in greater detail the technical implementation of the various components of the proposed architecture, as implemented in the experimental evaluation setup:

- Private blockchain: this blockchain was created and supported locally using Go Ethereum (geth) (version 1.10.3) [29] on the MQTT broker and the Smart contract proxy. The consensus protocol employed is Proof of Authority (PoA), since it is more resource-efficient than with alternative PoW [20] approaches. In this consensus protocol, signers are the nodes that are allowed to mine and validate blocks. Thus, they should be trustworthy nodes, which is why the MQTT broker and the smart contract proxy were assigned as signers. The protocol was configured to mine new blocks every 3 s, with the rest of the configurations assumed to be the default.
- Public blockchain: in order to promote more realistic results, the public blockchain was not implemented locally (e.g., via simulation of the Ethereum mainnet with *geth* nodes). Instead, the Ropsten Ethereum testnet was employed for this purpose. This approach was chosen because it is the testnet that is the most similar to Ethereum mainnet, given that it provides the same consensus protocol (PoW) and configurations. Moreover, since it is a testnet, its cryptocurrency is used for test purposes, meaning that the transactions and interactions with smart contracts do not have a real monetary cost. On the other hand, the mainnet (Ethereum) was not considered because it requires real ether, and as a result is considered to be out of the scope of the experimental evaluation study considered.
- MQTT broker: This VM is responsible for handling sensor controller requests received via MQTT. The MQTT broker server used for this purpose is HiveMQ, together with a python (version 3.8) script with the Web3.py library to use the geth instance to interact with the private blockchain. The python script also uses the libuplink-python library to communicate with the Storj storage system. Since it only works at the private blockchain level, it only runs one geth node instance. In terms of resources, 4 CPU cores and 3 Gb of ram were allocated for this purpose.
- Smart contract proxy: As mentioned previously, it is the middleware between the private and public blockchain. Therefore, this component is connected to both blockchains via two *geth* instances. This node is also running a python script in conjunction with *Web3.py* (version 5.19.0), which allows interaction with *geth* nodes to handle simultaneous requests from both blockchains (listen to events and execute smart contract functions). Since this VM handles the most resource-intensive tasks, 4 CPU cores and 4 Gb of ram were allocated.
- Agent: This VM works at the public blockchain layer; therefore, it runs only 1 *geth* node instance. Similarly to the two previous nodes, it also runs a python script with the *Web3.py* library to listen to events, and make data requests to smart contracts. The resources allocated for this purpose were 4 CPU cores and 3 Gb of ram.
- Sensor controller: this device uses a web application to communicate with the MQTT broker and process its requests. The user of the IoT application can define various ACL, as appropriate for the data types that he needs to handle and perform data management requests, such as access, modification or deletion. In this context, an add data option was added to the application, in order to simulate the vehicle’s behaviour, which was used to test the publishing of data, as well as for load testing. A direct blockchain communication option was also implemented in our application, using the *Web3.js* (version 1.3.6) API, together with a *geth* instance. This alternative communication method allows us to compare alternative communication approaches with the blockchain, either directly or via the MQTT middleware. A cellular network emulator was also employed [30], to provide a more realistic testing environment. This emulator consists of a NetEm (Network Emulator) [31] profile to emulate mobile data communications in 4G network environments with roaming. The profile used imposes a delay distribution on the network interface, which emulates the connectivity latency of the cellular access network. As this VM represents a constrained device, we have considered the allocation of 1 processor with 2 Gb of RAM to support the previous functionalities.

The tests were run with a fixed duration of 300 s, and considering various requests rates, in particular 1, 10 and 50 req/s. This allows us to test the system’s performance in various load scenarios. Each one of the test runs was executed five times, in order to build a more representative result sample, while also contributing to reduce experimental errors. The requests used for the performance tests were the *publish data* in the sensor controller, and the *get data* in the agent node. We note that these requests simulate the submission of data by smart vehicles in the context of the application (by using *publish data*), as well as the request of data (using *get data*) by public nodes (agents and users), which represent the majority of requests expected to be performed in a real implementation of the system. Therefore, it is considered the most relevant in order to ascertain the performance and scalability of the proposed approach.

In the sensor controller, two communication methods were considered: MQTT and direct blockchain communication. As previously discussed, the NeTem profile was employed to emulated 4G connectivity with good cellular coverage and roaming, in order to simulate a mobile environment where devices communicating with the infrastructure roam between different cellular towers.

### 4.2. Evaluation Metrics

To evaluate the viability of the proposed approach, a set of metrics was considered in our experimental evaluation study, as we proceed to discuss in detail:

- Time overhead: obtained using the python’s *time* library, used to measure the time required to handle particular requests. We also note that the sensor controller *publish data* request results are dependent on clock synchronisation with the MQTT broker, which can add a 1–2 s error. Despite the experimental evaluation scenario being limited in terms of computational resources, this metric allows inferring on the performance of the system.
- Bandwidth consumption: this was quantified using NetHogs (version 0.8.6) in Kbps, which allows measuring the bandwidth used by each process running in the operating system. This metric was measured in the sensor controller, for comparison between the two communication approaches. The goal in this evaluation is to obtain the network overhead of different communications approaches, in particular via MQTT and direct blockchain communications, and consequently be able to assess which is the most lightweight approach.
- CPU and memory usage: such resources were measured in each of the system nodes (in %) using the *psutil* (version 5.8.0) python library. Similarly to the previous metrics, it measures the computational overhead, to infer how the system behaves in congested scenarios.

### 4.3. Result Analysis

We next analyse the results obtained in our experimental evaluation study of the proposed architecture. We start by analysing results related to the transmission of the request *publish data* between the sensor controller and the MQTT broker, to infer what is the most beneficial communication method for resource-constrained environments, in particular MQTT communication (the default scenario) versus direct blockchain communications between the sensor controller and the PBVU (the considered alternative approach). We next proceed to analyse the system’s overall performance, with the goal of verifying its current performance and identify future work that can be considered to improve the performance and scalability of the proposed approach.

#### 4.3.1. Alternative Communication Approaches in the Sensor Controller

Table 1 illustrates the results obtained in the evaluation study performed to compare MQTT against direct blockchain communications. The table also considers two independent variables: request frequency rate (in req/s) and the communication method employed. In this table, we also show the median and standard deviation results for the 5 runs performed under each test condition. The first scenario corresponds to the default communication procedure explained in Section 3.6.3, in which the sensor controller uses the MQTT broker as an intermediary with the blockchain to handle its requests. In the alternative communication scenario, the sensor controller communicates directly with the blockchain, using Web3 and a geth node.

We may observe a clear difference between the two technologies, with blockchain being more resource-intensive than MQTT for all the considered metrics and test loads. We may thus observe that MQTT is a more lightweight approach, which is beneficial in particular for IoT applications employing resource-constrained devices. Furthermore, in the context of this evaluation, a statistical analysis was considered to provide a rationale for our conclusions. Since in our evaluation the samples do not comply with the assumptions to perform a parametric test (homogeneity of variance and normality [32]), a non-parametric two-way ANOVA alternative was considered, in particular, the randomisation test with unrestricted permutations [33]. This test examines three null hypotheses simultaneously, namely: no difference in means due to the communication method, no difference in means due to the request rate and no interaction of factors. The statistical test was performed for each dependent variable (bandwidth consumption, CPU usage and memory usage) with a significance level of 5%. The obtained *p*-value, in all hypotheses, was less than 5%, which rejects all the null hypotheses. Thus, the assumption that the MQTT communication method is more lightweight than blockchain is sustained in our analysis.

**Figure 9:**
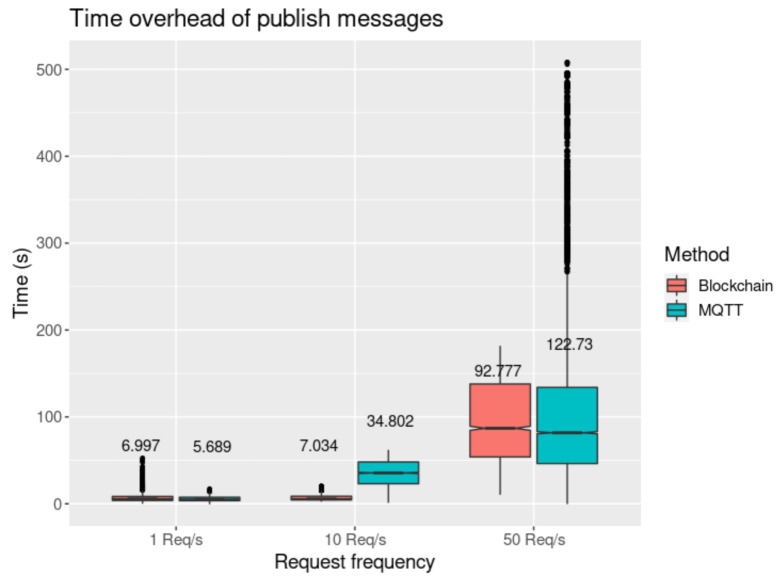
Time overhead measured in the sensor controller with mean values.

Even though MQTT requires less computational power, it adds more time overhead to the requests, and the results have more variability, as the frequency of the requests grows (Figure 9). This behaviour occurs due to the extra layer of latency and queuing required when using MQTT, making the time overhead results more bursty, as is visible in particular for a rate of 50 req/s.

In our following analysis, we focus on the evaluation of the performance of the proposed approach. This allows us to ascertain not only the viability of the architecture itself, but also to infer its scalability. As we discuss at the end of the article, future work can also be conducted in order to implement further mechanisms in the context of the proposed architecture, which will contribute to promote its resiliency and scalability.

#### 4.3.2. Performance Evaluation

**Figure 10:**
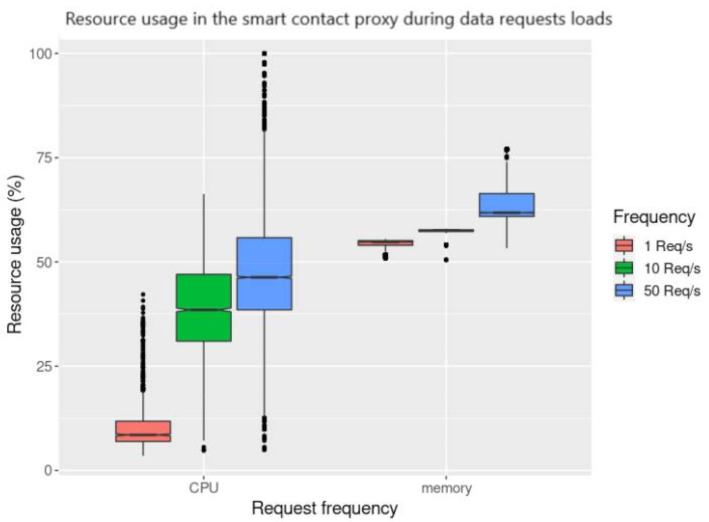
Resource consumption measured in the smart contract proxy with mean values.

**Figure 11:**
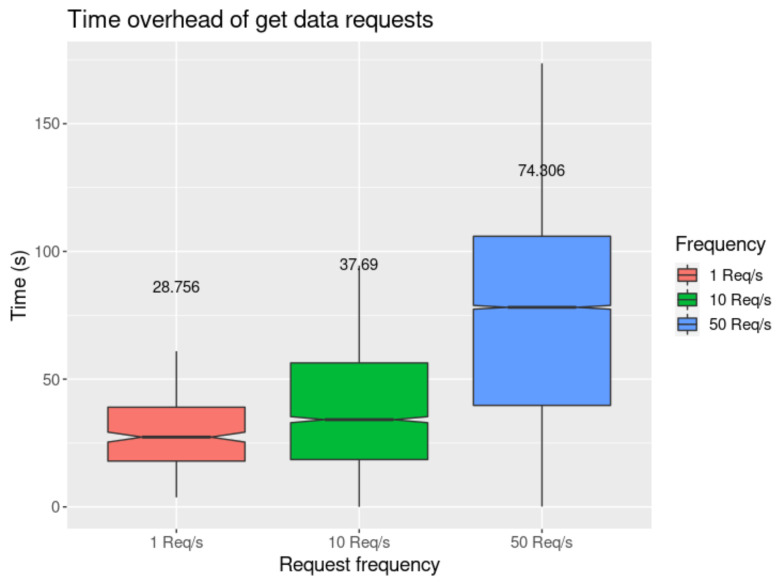
Time overhead measured in the agent with mean values.

In Figure 10 and Figure 11, we present the results obtained when using the *get data* request tests, performed in the agent node (the requester) and smart contract proxy (the handler). This process is detailed in Section 3.6.4 and depicted in Figure 6. The values were measured starting when the requests are sent until a response is received. We may observe that there is a distinctive difference in time overhead between these requests and the *publish data* requests illustrated in Figure 9. The private blockchain offers much lower latency, with the mean result for 1 req/s to the private blockchain having been measured at 5.689 s (using MQTT), and 6.997 s with direct blockchain communications. In the public blockchain, it has been measured at 35.085 s. We also observe that for the *get data* request, performed in the public blockchain and requiring two transactions to be completely handled (request and response), the mean of each transaction is 17.543 s, which is approximately two times higher than in the private blockchain.

**Figure 12:**
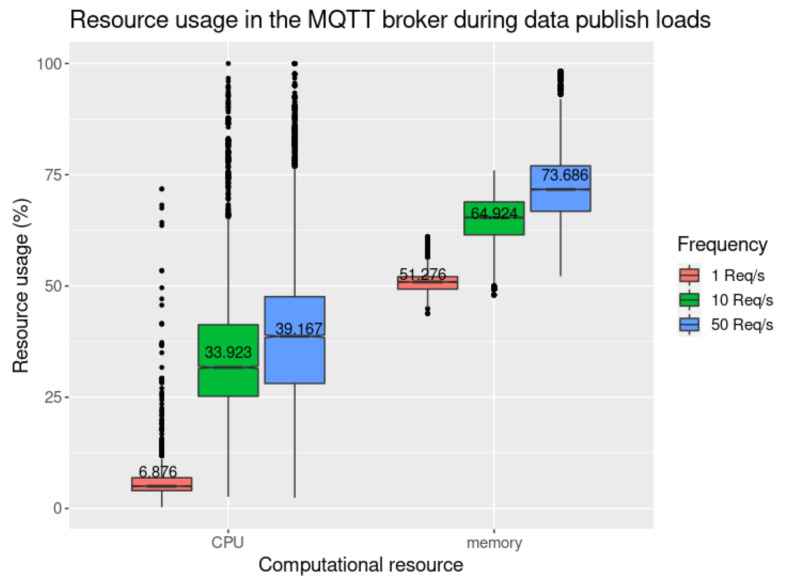
Resource consumption measured in the MQTT broker with mean values.

By analysing Figure 10 and Figure 12, we may note that both request handlers (smart contract proxy and the MQTT broker) follow the same pattern. In particular, as the request rate increases, the resource consumption also increases, the same applying to the variability of the measured results. In this specific scenario, we can infer that the system’s throughput is close to 50 req/s, given that the time overhead has much more variability, and the resource consumption reaches maximum capacity on particular occasions.

We should also note that it is excepted that the performance of the Ethereum private blockchains will increase, as more computational resources are added to the system [34]. However, from our experimental evaluation, we were able to observe the behaviour, as well as the limitations, of our experimental implementation scenario. Despite the visible impact of the blockchain, it nevertheless does not prevent its usage to support security and privacy.

It is important to note that in the experimental evaluation of the proposed architecture, we consider what is, fundamentally, a proof of concept implementation. In future work, a cloud implementation will be employed supporting an optimal setup environment, in the context of which vertical and horizontal elasticity, as well as redundancy mechanisms, may be enabled. In this context, we realise that, in its current form, some architectural components employed may prove to impose some limitations. In particular, the MQTT broker and the smart contract proxy are single points of failure, given that currently such entities are not supported by resilience mechanisms. The blockchain itself also has an impact on the throughput of the system, since the consensus protocol enforces a limit on the number of transactions that are processed per second. Optimisations can also be added to the software developed. Finally, we recognise that the emulators used in the evaluation scenario can also impact by adding to overall latency, an aspect we plan to address in future developments in the implementation of the proposed architecture. Despite such limitations, we consider that the experimental evaluation study of the proposed architecture herein described, provides sufficient data to ascertain the viability of the proposed approach.

### 4.4. Security and Privacy

Our evaluation of the proposed architecture in the light of the considered security and privacy requirements was supported by the logs generated by the various entities of the system, as such information allowed us to verify and validate the correct implementation and operation of the functionalities related to such requirements. The evaluation of these requirements is summarised in Table 2.

Overall, we may conclude that the system manages to deliver high privacy and security to its users and data. In particular, the tiered architecture of blockchains strengthens the privacy and security in our approach, since the private blockchain can be used to store information privately, while the public blockchain is used to exchange information in a secure and “immutable” approach. Furthermore, we use the addressing system of blockchains to anonymise the user throughout the whole system, thus enhancing its privacy. In addition, MQTT also allows the implementation of privacy and security measures through authentication and authorisation. Finally, the usage of Storj enhances the privacy and security of the users’ data and allows data management procedures to increase the compliance of the system with the privacy requirements previously identified.

In conclusion, we may observe a clear compromise between privacy and performance, mostly due to the fact that blockchain is still a technology in development, and in particular due to its limited transaction throughput, which is one of its major drawbacks. Despite this, blockchain brings important advantages in what respects the fulfilment of critical security and privacy requirements which, in our opinion, clearly compensate its impact on performance. As previously discussed, our conclusions do not exclude the opportunity to evolve the implementation of the proposed architecture in the future, therefore leaving room for various types of optimisations. We address such opportunities for future work at the end of the article, while in the next section we focus on related work.

## 5. Related Work

A panoply of privacy-preserving proposals for IoT are present in the literature. We may observe that some of these works focus on improving certain technologies, to be adequate for constrained environments, such as lightweight cryptography [19,35] or enhancing the security of current technologies [3]. An alternative approach is to address privacy by design, and, in this context, blockchain can aid in securing IoT applications, as we have addressed in the article.

In our proposal, MQTT is used as a complementary technology to improve interoperability between constrained IoT devices and the blockchain and reduce computational resource requirements, whereas in the proposal of [5] blockchain is used as a complementary system to offer a more reliable authorisation and authentication approach, using smart contracts, applied to MQTT. Furthermore, our system’s users are not bound to only a system, but both blockchain and MQTT, since their blockchain addresses are also used to identify them in the MQTT’s authentication and authorisation mechanisms.

The proposal in [7] also targets mobile IoT applications, in particular for the automotive sector, and is also user-centred. The user can decide the data that he wants to share and to whom; he is also aware of the data being transferred and which data is stored. However, our solution offers privacy control and data management functionalities to the user, such as the deletion of data or its modification, which is not supported in [7]. This property is also lacking in other works ([8,9]), wherein the data is stored directly in-chain, and, therefore, cannot be managed due to the tamper resilient nature of blockchains.

To solve the in-chain storage problem, data is stored off-chain in [4,7,10]. In [10], the integration of an external decentralised storage system is proposed, in particular the Interplanetary File System (IPFS). Similarly to our proposal, data can be accessed in-chain through an access serial, in this case, IPFS hash stored in a smart contract. Although the data is being stored in an external solution, IPFS does not support guaranteed modification nor deletion of data, which is a limitation of the system. Our approach is thus distinguished by the fact that we implement Storj to mitigate this issue, as well as to support GDPR policies such as data erasure, as previously discussed.

Contrary to our work, the IoT devices in [4] communicate directly with the blockchain, which can be a computational overhead for the devices, as we discussed in Section 4.3.1.

As previously observed, existing proposals also do not focus on user-controlled privacy, as per the goals of our proposal. As discussed throughout the article, user-controlled privacy is a fundamental enabling aspect of many applications, and one that is becoming fundamental, also from a legal perspective [1].

## 6. Conclusions and Future Work

The proposed architecture can certainly evolve in the light of future privacy, security and functional requirements, which can bring more functionalities and solidify its security and user-controlled privacy mechanisms. In this context, we may consider various future research opportunities, as we proceed to discuss.

One is to design privacy-preserving mechanisms to allow the public nodes to identify the data they can access, without disclosing the ACL of the data entry or other private information. Reputation systems can also be considered to identify misbehaving nodes, which may be required, for example, to pay a higher fee for each request. The transparent nature of blockchains can also be of help to keep track of the nodes that request access to data while informing data owners (e.g., via logs).

A possible approach can also be to use blockchain to manage the node’s public keys, for example via a smart contract for the support of integrity. High availability mechanisms may also be investigated, for example, to support load balancing between brokers, smart contract proxies and the PBVU entities of the proposed architecture, thus removing single points of failure in a future evolution of our system implementation.

Regarding the usage of blockchain, other implementations can also be considered and evaluated (e.g., Cardano [23] and Solana [36]), that may offer more throughput and other functionalities. In addition, our system could be implemented in a cloud environment, since it may help in supporting further availability mechanisms and more performance, also due to the inherent horizontal elasticity of such environments. A future setup with such goals could provide an alternative environment for experimental testing and performance analysis, as previously discussed. In order to have a more complete system, a mechanism with policies or rules can also be implemented in order to add new nodes into the system, in particular, in the PBVU. In addition, optimisations of code can also be explored, such as the smart contracts, to take into consideration the gas fees.

IoT is a notable technology that is becoming more embedded in our daily lives. Thus, implementing solutions that preserve the privacy of its users and data in line with legal aspects such as those described in GDPR is becoming a fundamental goal. In this article, we propose a blockchain-based architecture that focuses on providing user and data privacy for mobile IoT applications. In our proposal, we leverage the power of blockchain to support access control, provide anonymity to users, and provide a common channel for heterogeneous systems to exchange and request information. Additionally, blockchain is employed in tandem with other technologies, in particular MQTT and Storj, to mitigate some of the limitations that emerge in these kinds of applications that use constrained devices and operate with sensitive information.

In conclusion, the proposed architecture paves the road for the support of a robust privacy-preserving solution, not only for automotive applications, but also for other IoT applications that require security and user-controlled privacy. 

## Figures and Tables

**Table 1:** Resource consumption results (measured in the sensor controller) for alternative communication approaches.

Rate	Communication Method	Bandwidth Consumption (Kbps)/Standard Deviation	Mean CPU Usage (%)/Standard Deviation	Mean Memory Usage (%)/Standard Deviation
1 req/s	MQTT	0667/0148	5367/1629	14,456/0063
	Blockchain	4073/1965	10,252/7268	69,191/2440
10 req/s	MQTT	7580/7813	8233/4887	15,048/0435
	Blockchain	14,744/8654	30,689/21,507	96,098/3360
50 req/s	MQTT	13,217/10,198	12,853/6120	15,817/0444
	Blockchain	64,100/28,767	29,997/20,302	98,261/2032

**Table 2:** Validation of security and privacy requirements.

Requirement	Evaluation
User controlled privacy	One of the GDPR policies dictates that the user should be able to specify their privacy preferences over their data. However, in most classical applications and proposals ([4,8,9,10]), the user is unable to define their privacy preferences, which could result in data being used by other entities without their consent. In our proposal, the user has the freedom to specify the list of public nodes that can access their data, which thus enhances their privacy.
User anonymity	Applications frequently gather information about the user for KYC purposes or other goals. Even if this information is being used for legitimate purposes and has the user’s consent, it still opens avenues to potential attacks. Therefore, implementing enhanced anonymity by design could mitigate such privacy threats, which makes the blockchain’s addressing system beneficial to improve the overall privacy of users in the system. In particular, we note that it is only possible to know to whom the address belongs to if the owner advertises it.
Location and data privacy	Other proposals store data in the public blockchain [9], which imposes a threat to data privacy since it is publicly available to the public nodes or in other storage systems that do not guarantee its security (e.g., in centralised storage systems). Thus, storing data off-chain and in a decentralised system (as with Storj) improves the overall privacy and security of data. Furthermore, inherently from the user anonymity, the location information and data generated by smart vehicles is anonymous across the whole system.
Data confidentiality	The data is encrypted with symmetric cryptography when it is in transit or stored in Storj, and can only be decrypted with the specific symmetric key. Thus, the confidentiality of the data is guaranteed.
Authentication and Authorisation	The Role-Based Authentication Control (RBAC) plug-in integrated into MQTT supports user authentication and authorisation. Each user has to be pre-registered in the database to authenticate himself, and various roles can be created to restrict the operations allowed to each particular user, e.g., a vehicle user can only publish or subscribe to the topic that corresponds to its blockchain address.

## Data Availability

The datasets generated during the study are available at https://github.com/KevinCar98/IoT-BlockchainBased, accessed on 29 June 2021.
